# Pediatric Gaucher Disease Type 3 Presenting with Oculomotor Apraxia: A Case Report

**DOI:** 10.3390/children11080960

**Published:** 2024-08-09

**Authors:** Margherita Di Costanzo, Nicoletta de Paulis, Giuseppe Cannalire, Nicola Morelli, Giacomo Biasucci

**Affiliations:** 1 Pediatrics and Neonatology Unit, Guglielmo da Saliceto Hospital, 29121 Piacenza, Italy; n.depaulis@ausl.pc.it (N.d.P.); g.cannalire@ausl.pc.it (G.C.); giacomo.biasucci@unipr.it (G.B.); 2 Diagnostic Neuroradiology Unit, Department of Radiological Functions, Guglielmo da Saliceto Hospital, 29121 Piacenza, Italy; n.morelli@ausl.pc.it; 3 Department of Medicine and Surgery, University of Parma, 43126 Parma, Italy

**Keywords:** lysosomal storage disorder, beta-glucocerebrosidase, enzyme replacement therapy, splenomegaly, inherited metabolic diseases

## Abstract

We report on a 4-year-old boy affected by Gaucher disease (GD) type 3, who presented with splenomegaly and a history of oculomotor apraxia. GD is a rare lysosomal storage disorder caused by glucocerebrosidase deficiency with multi-organ involvement. Besides common clinical features such as hepatosplenomegaly and skeletal involvement, less frequent neurological symptoms, such as oculomotor apraxia, are indicative of neuronopathic forms of the disease, namely GD type 3, to be confirmed both by enzyme activity and genetic testing. Overall, GD management requires a multidisciplinary approach involving metabolic pediatricians, neurologists, psychologists, and geneticists, and currently relies on early enzyme replacement therapy. Although enzyme replacement therapy has proved to be effective in improving systemic signs and symptoms, it is unable to alleviate neurological complications once these have occurred, as it does not pass across the blood–brain barrier. Neurological improvements may occur through indirect mechanisms. Thus, our case report aims to highlight the importance of considering GD in the differential diagnosis of pediatric patients presenting with splenomegaly associated with neurological manifestations, as early intervention may significantly modify the disease progression and prevent further irreversible complications.

## 1. Introduction

Gaucher disease (GD) is a rare inherited lysosomal storage disorder. The worldwide incidence of GD varies across different countries, ranging from approximately 0.39 to 5.80 per 100,000 individuals. GD is an autosomal recessive disorder due to a congenital enzymatic deficiency of glucocerebrosidase, which is responsible for breaking down glycosphingolipids in the lysosomes. The accumulation of glucocerebroside and other glycolipids in multiple tissues can cause damage to various organ systems. GD typically presents in childhood with symptoms such as bruising, bleeding, fatigue, and bone pain, or a combination of these [1,2]. There are three main phenotypes of GD: type 1 (chronic, non-neuronopathic), type 2 (acute neuronopathic), and type 3 (chronic neuronopathic) [1]. Children with GD type 1 typically exhibit splenomegaly, hepatomegaly, anemia, thrombocytopenia, poor growth, pubertal delay, and acute and chronic pain related to bone involvement. GD type 1 usually manifests in childhood. GD type 2 is characterized by early severe neurological symptoms, while GD type 3 has a slower progressive neurological involvement. GD type 3 presents the same visceral manifestations as GD type 1 but in a more severe form. Patients with GD type 3 develop neurological symptoms over time, such as cognitive impairment, myoclonic seizures, ataxia, spasticity, and muscle weakness [1,2,3].

In patients with GD type 3, neurological findings mainly include oculomotor apraxia. As evidenced by a recent literature review conducted by Kurolap and colleagues, only seven patients (20%) affected by Gaucher disease type 3c have been described without oculomotor findings [4].

Nevertheless, the occurrence of oculomotor apraxia as the initial or isolated neurological manifestation of Gaucher disease is rare [5]. In their literature review, Kurolap and colleagues reported only nine cases of GD type 3c with oculomotor apraxia [4]. Oculomotor apraxia is defined as impaired volitional horizontal eye movements. It is often accompanied by compensatory lateral head thrusts. The clinical course and extra-neurological organ involvement aid clinicians in the differential diagnosis.

Herein, we report on a child presenting with splenomegaly and a history of oculomotor apraxia, who was subsequently diagnosed with GD type 3.

## 2. Case Report

K., a 4-year-old boy, was admitted to our Pediatric Emergency Department for arthralgia. As he presented with left wrist swelling, an X-ray was performed, showing no fractures or other bone lesions. According to the orthopedic evaluation, a cast was then applied. Due to fever and tonsillitis, a rapid throat swab for Group A β-hemolytic Streptococcus was performed, which tested positive, thus requiring oral amoxicillin therapy. Within the following 72 h, the patient was admitted to our Pediatric Emergency Department twice due to the persistence of symptoms affecting the left wrist joint and the appearance of swelling, functional impotence, and pain in the left ankle. On his third visit, K. was hospitalized for further evaluation and treatment. On admission to our Pediatrics Unit, K. was afebrile and in good general clinical condition, with pinkish skin color, preserved hydration status, and regular cardiorespiratory parameters. Heart tones were valid and rhythmic, without any pathological murmur, with a 110 bpm heart rate. A physiological vesicular murmur was heard in the entire lung area. The patient’s peripheral oxygen saturation rate was 99% in ambient air. The pharynx was hyperemic with tonsillar hypertrophy. He reported painful swelling in both ankles and mild pain and swelling in the left wrist despite the cast. The abdomen was treatable and painless upon examination, but the spleen size was found to be remarkably enlarged; liver size was within normal limits.

### Personal History

K. was born at term via vaginal delivery; his birth weight (3390 g) was adequate for gestational age. The postnatal course was regular, as well as his psychomotor development throughout infancy. However, at the age of 1 year, his parents reported the occurrence of sudden head movements, referred to as ‘jerking’, mainly in the extreme left gaze and occasionally in the right gaze. When he was 2 years old, he underwent his first ophthalmologic/orthoptic evaluation, which revealed horizontal paralysis of the left gaze, which was more evident than the right one, with no signs of paralysis of the extrinsic eye muscles. The diagnosis of oculomotor apraxia was then made at the age of 3 years. A brain MRI was performed and found to be normal. Additionally, his neuropsychomotor development was age-appropriate. No family history of neuropsychiatric disorders or ophthalmological disease was reported. During his hospital stay, K. was given anti-inflammatory therapy with oral ibuprofen. Additionally, the antibiotic therapy with amoxicillin was continued. Blood tests showed a normal blood count, with slightly increased inflammatory markers, such as erythrocyte sedimentation rate (ESR) of 68 mm/h (normal range: 2–38 mm/h) and C-reactive Protein (CRP) of 1.5 mg/dL (normal range: 0–0.5 mg/dL). Due to a sharply elevated (2667 IU/mL; normal range: 0–200 IU/mL) anti-streptolysin-O (ASO) titer, specific clinical-instrumental tests were performed to rule out the diagnosis of rheumatic fever. As the cardiac ultrasound scan and electrocardiogram were normal, the criteria for the diagnosis of rheumatic fever were not fully met. Moreover, due to the concomitant presence of splenomegaly, a complete ophthalmological examination was performed to assess the potential onset of Juvenile Idiopathic Arthritis, but no signs of ongoing uveitis and/or iridocyclitis were present. Additionally, the rheumatoid factor (RF) and antinuclear antibodies (ANA) were both negative. To rule out infectious causes of arthritis, we performed serological tests for Cytomegalovirus, Toxoplasma, Epstein–Barr virus, Herpes simplex 1–2 virus, and Bartonella, all of which tested negative. An abdominal ultrasound scan confirmed a significantly enlarged spleen with a maximum pole–polar diameter of approximately 13 cm and a transverse diameter of 5 cm, without any other anomalies. The child’s painful symptoms and joint swelling regressed; hence, he was discharged with an oral ibuprofen prescription for an additional 10 days. At the end of the prescribed therapy, the joint symptoms regressed, and the ASO titer decreased to 1641 IU/L.

In view of the clinical history reporting head movements that might have been considered as a “tic syndrome”, as well as the oculomotor apraxia, the diagnosis of PANDAS (pediatric autoimmune neuropsychiatric disorders associated with streptococcal infections) should have been ruled out by testing the anti-DNAse B (anti-deoxyribonuclease-B) antibody titer. Indeed, PANDAS may be considered as part of the larger category of the pediatric acute-onset neuropsychiatric syndrome (PANS), which includes acute behavior change with obsessive–compulsive disorders, anxiety, emotional lability, depression, irritability or oppositionality, sensory-motor abnormalities, and several somatic symptoms, with skin, ocular, gastrointestinal, musculoskeletal, and neurological involvement [6].

**Figure 1:**
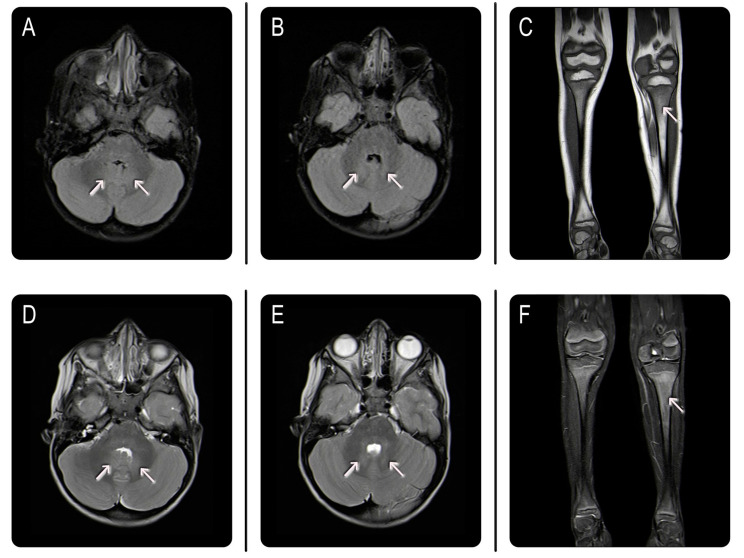
Brain and lower limbs MRI: (**A**,**B**) axial fluid-attenuated inversion recovery (FLAIR) and (**D**,**E**) T2-weighted Fast Spin-Echo (T2W-FSE) sequences revealed abnormal patchy hyperintense lesions (white arrows) in the dentate nuclei of the cerebellum at different levels. On coronal T1-weighted (**C**) and T2W-FSE (**F**) images, a subtle area of hypointensity signal was evident at the level of left tibia diaphysis (white arrow), finding that suggested bone marrow infiltration.

However, our patient’s brain MRI picture (see below in the text) showed anomalies in the dentate nucleus of the cerebellum, which are not as suggestive of PANDAS/PANS as anomalies involving the basal ganglia are. Based on this specific finding, and due to the absence of other clinical features suggestive of PANDAS, we decided not to perform the anti-DNAse B test; rather, the child’s clinical signs and symptoms, the previous history of oculomotor apraxia, and the exclusion of other possible causes due to the inconclusive investigations performed brought us to further investigate possible underlying inherited metabolic diseases. Among these, we tested for lysosomal storage diseases, such as Mucopolysaccharidoses, which were ruled out, and GD, which was confirmed by a markedly reduced acid β-glucocerebrosidase activity on a Dried Blood Spot (DBS) and by the presence of a heterozygous c.754T > A mutation (inherited from the father) and c.882T > G and c. 1342G > C mutations (inherited from the mother) on the GBA gene. These mutations had previously been reported as pathogenic for GD. The combination of these gene variations with the child’s clinical features led to the final diagnosis of GD type 3, which is also known as juvenile or subacute neuronopathic form. A brain MRI was then repeated, showing a shaded FLAIR hyperintensity in the anatomical region of the dentate nuclei, consistent with the patient’s medical history and clinical picture of GD type 3. Lower limbs MRI revealed shaded hypo-intense signal alteration at the level of the proximal and distal metaphyses of the femurs bilaterally, compatible with the persistence of red marrow. A subtle area of hypointensity signal on the T2 weighted image was evident at the level of the proximal femur metaphysis and left tibia diaphysis, which is compatible with medullary infiltration related to the patient’s disease. There were no signs of osteonecrosis, pathological fractures, or bone infarction (Figure 1).

The patient was immediately scheduled for enzyme replacement therapy (ERT) via intravenous imiglucerase (Cerezyme^®^, Sanofi B.V., Paasheuvelweg 25, 1105 BP Amsterdam, The Netherlands), infusion, so far provided at 60 IU/Kg/dose fortnightly in our Pediatrics Unit, which is a Regional Referral Clinical Center for the diagnosis, therapy, and follow-up of Inherited Metabolic Diseases.

A specific follow-up protocol was then scheduled, including blood chitotriosidase, lysoGb1, ferritin, acid phosphatase, and ACE assays, in addition to a complete metabolic–nutritional routine and a complete abdominal ultrasound. After 6 months from the start of ERT, the patient showed a good height–weight recovery and improved nutritional blood tests (Table 1 and Table 2).

At diagnosis, the upper abdominal MRI revealed the presence of hepatosplenomegaly, with cranio–caudal diameters of approximately 12 cm for the liver and 14 cm for the spleen, respectively, in the absence of evident focal or diffuse intraparenchymal alterations.

The abdominal ultrasound performed during the follow-up examination confirmed the presence of homogeneous splenomegaly, which was found to be substantially stable. The maximum pole–polar diameter was approximately 13 cm, while the transverse diameter was approximately 5 cm. The liver was observed to be of maximum overall size, situated at the upper limits of the norm, with a homogeneous echo density and clear margins.

At the follow-up eye examination, there was evidence of improved compensation of oculomotor apraxia by head movements.

The patient’s follow-up is ongoing, and the blood chemistry and instrumental tests (such as abdominal ultrasound and brain and lower limbs MRI) will be repeated 12 months later.

## 3. Discussion

This case report describes a 4-year-old pediatric patient diagnosed with GD type 3 after presenting with pronounced splenomegaly and osteoarticular symptoms. The presence of splenomegaly and the patient’s medical history of oculomotor apraxia, diagnosed at 3 years of age, raised suspicion of GD. The diagnosis was confirmed by observing a significantly reduced acid β-glucocerebrosidase activity on a DBS, alongside the detection of heterozygous mutations: c.754T > A inherited from the paternal lineage and c.882T > G and c.1342G > C inherited from the maternal lineage, both localized on the GBA gene.

Oculomotor apraxia is a rare but significant manifestation of GD, particularly in more severe neuronopathic forms, such as type 3. It is characterized by difficulty in voluntary eye movement, which may result from the accumulation of lipid substrates in the central nervous system, including the brainstem, affecting the coordination of eye movements [3,7]. Splenomegaly is a common feature of GD, resulting from the accumulation of globotriaosylceramide (GL-1) in the spleen cells. This symptom can be an early sign of the disease and may lead to complications such as anemia, thrombocytopenia, and splenic dysfunction [3].

Bone involvement is a common feature in GD, reported in approximately 75% of patients [3]. Manifestations of bone involvement include bone infarcts, avascular bone necrosis, lytic lesions, osteosclerosis, fractures due to osteopenia or osteoporosis, and seldom acute osteomyelitis. Impaired mobility, poor performance status, and increased morbidity may result from even intense bone pain, fractures, and progressive joint collapses [8]. Reduced bone mass can be diffuse or localized and may affect both cortical and trabecular bone. It has been shown to occur next to the sites of Gaucher cell infiltration. Failure to achieve optimal bone mass is likely to affect peak bone mass and may contribute to osteoporosis, thus increasing the risk of pathological fractures and joint collapse during adolescence. Additional prospective data are needed to refine treatment strategies for primary fracture prevention in children with GD and bone fragility in order to prevent significant related morbidity.

The advent of novel approaches to the diagnosis and treatment of GD has the potential to markedly improve patient outcomes. Advances in diagnostics include the use of next-generation sequencing (NGS) for rapid and accurate identification of GBA gene mutations, as well as the emergence of new biomarkers such as chitotriosidase, chemokine CCL18, and more recently, glucosylsphingosine (lyso-Gb1) [9]. These provide non-invasive diagnostic tools and enable the monitoring of treatment efficacy.

In terms of therapeutic intervention, ERT with imiglucerase, velaglucerase alfa, or taliglucerase alfa still represents the primary approach for addressing systemic symptoms [10]. However, its efficacy in managing neurological symptoms remains constrained. Nevertheless, although ERT with glucocerebrosidase does not usually cross the blood–brain barrier due to its large molecular size, there have been reports of some neurological improvements in selected patients, including our patient. Neurological improvements may occur through indirect mechanisms. ERT reduces glucocerebroside accumulation in peripheral tissues, thereby decreasing systemic inflammation and oxidative stress, which may have beneficial effects on the central nervous system. Additionally, reduced activation of peripheral immune cells can lower central nervous system inflammation. ERT may also enhance peripheral nervous system function, influence the production of neurotrophic factors, and modulate genetic and epigenetic pathways that indirectly support neuronal health.

New formulations and delivery methods are currently being developed with the aim of improving efficacy and patient compliance. One area of research that has attracted attention is the potential for oral ERT.

Substrate reduction therapies, such as eliglustat, have demonstrated efficacy in reducing substrate accumulation in GD, offering a valuable oral alternative for adult patients who prefer this route over intravenous infusions. Eliglustat has shown encouraging outcomes in alleviating systemic symptoms; however, its efficacy in addressing neurological symptoms remains a subject of ongoing investigation [10].

Gene therapy should represent the ultimate approach for the correction of the underlying genetic defect in GD. Preclinical studies employing lentiviral vectors and CRISPR-Cas9 gene editing have yielded encouraging results, suggesting the potential for long-term correction of enzyme deficiency [11,12].

Pharmacological chaperone therapies, such as ambroxol, are designed to stabilize the defective glucocerebrosidase enzyme, thereby enhancing its function [13].

Regarding neurological complications, it is of the utmost importance to conduct regular neurological assessments and utilize advanced neuroimaging techniques for the early detection and management of patients with GD type 2 and type 3. The current research aims to find neuroprotective strategies, including antioxidants, anti-inflammatory drugs, and other agents enhancing neuronal survival and function, to prevent or mitigate the onset and progression of neurological damage [14].

Lastly, hematopoietic stem cell transplantation has been employed in severe cases of GD with neurological complications, offering a potential permanent source of healthy cells producing functional glucocerebrosidase [15,16]. However, this approach is associated with significant side effects and collateral risks.

## 4. Conclusions

The broad range of clinical manifestations of GD underscores the importance of comprehensive evaluation and targeted management to improve patients’ outcomes.

A multidisciplinary approach is crucial for effective management, requiring close cooperation among specialists from diverse areas such as metabolic medicine, pediatrics, neurology, ophthalmology, psychology, and genetics. This collective effort is crucial in addressing the diverse clinical manifestations and complexities associated with GD type 3, particularly in the cases presenting with neurological involvement, such as oculomotor apraxia.

ERT is a cornerstone for the treatment of GD, offering a targeted approach to mitigate the underlying enzyme deficiency and alleviate systemic manifestations. Though ERT is not able to pass across the blood–brain barrier, it has been shown to be of utmost importance also for patients with GD type 3 for their often devastating visceral symptoms and significant hematological abnormalities. By reducing these signs and symptoms and, hopefully, by halting the progression of neurological symptoms, ERT should also be part of the standard care for patients with GD type 3, with the aim of improving their quality of life and longevity [17]. Close monitoring and individualized dosing regimens are necessary to optimize treatment outcomes and mitigate adverse effects. In our patient, after starting ERT, we observed an improvement in compensation of oculomotor apraxia by head movements, a good height–weight recovery, and improved nutritional blood tests. This case emphasizes the importance of routinely considering GD in the differential diagnosis of children with splenomegaly. Early initiation of ERT can positively affect the natural history of the disease and prevent serious complications.

## Figures and Tables

**Table 1:** Anthropometric parameters at diagnosis and after 6 months.

	At Diagnosis	After 6 Months	Unit	Percentiles * at Diagnosis	Percentiles * after 6 Months
Weight	17.9	19.850	kg	15–50^th^	50–85^th^
Height	108.5	112	cm	15–50^th^	15–50^th^
BMI	15.20	15.54	kg/m^2^	15–50^th^	50^th^

* WHO child growth standards.

**Table 2:** Blood markers of pathology and nutritional blood tests at diagnosis and after 6 months.

	At Diagnosis	After 6 Months	Unit	Normal Values
Chitotriosidase	684	232	µmol/h/L	8–121
LysoGb1	289.6	221.9	ng/mL	0–14
Acid phosphatase	28.4	14.8	U/L	<4.7
ACE	286	115	U/L	33–112
White blood cells	8.52	11.53	×10^3^/µL	4.00–10.00
Red blood cells	5.05	5.55	×10^6^/µL	4.00–5.40
Hematocrit	35.1	40.5	%	34–46
MCV	69.5	73	fl	82–98
MCH	21.6	24.3	pg	27–32
Hemoglobin	11.3	13.5	g/dL	12–16
Platelets	134	235	×10^3^/µL	150–450
Ferritin	90	66	ng/mL	12–120

## Data Availability

All the data are included in the manuscript.
